# Bevacizumab as a chemoprotectant: reducing oxaliplatin induced hepatic sinusoidal injury

**DOI:** 10.18632/oncotarget.26207

**Published:** 2018-10-09

**Authors:** Jonathan D. Mizrahi, Michael J. Overman

**Affiliations:** Michael J. Overman: Department of Gastrointestinal Medical Oncology, Division of Cancer Medicine, The University of Texas MD Anderson Cancer Center, Houston, Texas, USA

**Keywords:** hepatotoxicity, thrombocytopenia, oxaliplatin, bevacizumab, vascular endothelial growth factor (VEGF)

Oxaliplatin-induced hepatic sinusoidal injury (HSI) is a well-described toxicity from oxaliplatin-based chemotherapy. This toxicity can result in portal hypertension, splenomegaly and subsequent thrombocytopenia [1]. Oxaliplatin-induced HSI is dose-related and reversible. Histopathological changes characteristic of sinusoidal injury are seen and include endothelial microdissections, sinusoidal dilation, congestion and deposition of collagen in the perisinusoidal space [2].

Until recently evidence for this toxicity has been best documented during adjuvant therapy of stage II and III colorectal cancer (CRC) and during neoadjuvant therapy prior to hepatic resection for metastatic CRC [3, 4]. In a large cohort of stage II and III CRC patients treated with adjuvant FOLFOX, increases in spleen size were seen in 86% of patients with a spleen size increase of ≥ 50% in 24% of patients. Increases in spleen size correlated with a reduction in platelet count, *P* = 0.003 [3]. In the majority of cases the increase in spleen size returned to baseline within one year after the last oxaliplatin dose. Pre-operative oxaliplatin-based chemotherapy has also been correlated with histopathological changes of HSI in non-tumor bearing liver in CRC patients undergoing liver metastectomies. The development of oxaliplatin-induced HSI has been correlated with an increased morbidity from hepatic resection with an increased need for blood transfusions, biliary complications, and prolongation of hospital stay [5]. In the large randomized trial by the European Organization for Research and Treatment of Cancer (EORTC) assessing a short duration of preoperative FOLFOX chemotherapy (3 months) for resectable CRC liver metastases, neoadjuvant chemotherapy was deemed safe; however, post-operative complications were higher in the neoadjuvant chemotherapy arm, and one patient was unable to undergo surgery due to macroscopic liver damage [4].

Although the exact mechanism underlying this toxicity is not well established, it appears that oxaliplatin’s induction of reactive oxygen species with a resulting increase of VEGF-A plays a critical role [6]. In rat models of hepatic sinusoidal injury due to monocrotaline, a pyrrolizidine alkaloid, VEGF inhibition with either sorafenib or regorafenib demonstrated protection against this injury [7, 8]. In addition, in non-tumor bearing liver samples from patients undergoing oxaliplatin-based neoadjuvant therapy with and without bevacizumab prior to CRC hepatic metastectomies, bevacizumab appeared to reduce the extent of HSI in limited retrospective studies [3].

In order to evaluate the impact of bevacizumab on HSI and the relevance of HSI in a non-resectable metastatic CRC population, Overman *et al.* evaluated two large cohorts of metastatic CRC patients treated with oxaliplatin-based chemotherapy with and without bevacizumab [9]. Change in spleen size, as assessed on restaging computed tomography scans, was used as a surrogate for HSI and was correlated with changes in platelet counts. In an exploratory single institution retrospective cohort of 138 bevacizumab and 46 non-bevacizumab treated patients, the bevacizumab treated cohort demonstrated a longer median time to splenic enlargement (defined as ≥ 30% increase in size, *P* = 0.02) and a reduced rate of thrombocytopenia (< 150,000/mm^3^, *P* = 0.04).

However, more importantly, Overman *et al.* analyzed a second cohort of patients, randomly selected from the randomized multi-center phase III N016966 clinical trial investigating the addition of bevacizumab to fluoropyrimidine and oxaliplatin (FOLFOX or CAPOX) chemotherapy in frontline metastatic CRC patients. The two co-primary endpoints of the study were the time to development of splenomegaly (defined as ≥ 30% increase in size) and the time to thrombocytopenia (defined as platelet count < 100,000/mm^3^). Two-hundred patients were analyzed, of which 106 received bevacizumab and 94 received placebo. The time to development of splenomegaly was 7.6 months versus 5.4 months (*P* = 0.01) and the six-month cumulative incidence of thrombocytopenia was 19% versus 51% (*P* < 0.001) in the bevacizumab and placebo arms, respectively. Though a difference in grade 3 thrombocytopenia was not seen between groups, grade 2 thrombocytopenia (< 75,000/mm^3^ to ≥ 50,000/mm^3^) was statistically less common in the bevacizumab treated group (six-month cumulative incidence 4% versus 23%, *P* < 0.001). Interestingly, the greatest benefit was derived for the population of patients that had the largest baseline spleen size (*P* = 0.008).

This work adds to the growing literature surrounding the protective effect of bevacizumab on oxaliplatin-induced HSI and clearly demonstrates that this protective effect can be seen during the palliative treatment of metastatic CRC patients. In addition, this data further supports the clinical need to monitor changes in spleen size during oxaliplatin-based therapy in order to best understand changes in platelet counts. One limitation of this work is that the impact of bevacizumab upon chemotherapy dose intensity could not be determined as dose reductions were not directly ascribed to a specific toxicity in the N016966 trial. However, the N016966 clinical protocol did require a treatment delay in patients who developed a platelet count < 75,000/mm^3^, which suggests that patients on the bevacizumab arm may have had fewer treatment delays. The impact of such delays on treatment efficacy is unknown.

Further outstanding questions from this work exist. In particular, whether platelet thresholds for chemotherapy administration should vary depending on the presumed etiology of thrombocytopenia has not been defined. As bleeding risk is reduced in patients with thrombocytopenia resulting from splenic sequestration compared to those with underproduction, such recognition suggests the possibility of adjusting platelet thresholds for chemotherapy delays and dosing in patients with oxaliplatin-induced HSI [10]. In addition, this data supports further investigation into the use of anti-VEGF therapy in the neoadjuvant setting with regards to its role in reducing oxaliplatin-induced HSI prior to CRC liver resection.
